# Low threshold unmyelinated mechanoafferents can modulate pain

**DOI:** 10.1186/s12883-017-0963-6

**Published:** 2017-09-15

**Authors:** Kathrin Habig, Anne Schänzer, Wolfgang Schirner, Gothje Lautenschläger, Benjamin Dassinger, Håkan Olausson, Frank Birklein, Elke R. Gizewski, Heidrun H. Krämer

**Affiliations:** 1 Department of Neurology, Justus Liebig University, Klinikstr. 33, 35392 Giessen, Germany; 2 Institute of Neuropathology, Justus Liebig University, Arndtstr. 16, 35392 Giessen, Germany; 3 Department of Radiology, Justus Liebig University, Klinikstr.33, 35392 Giessen, Germany; 4 Center for Social and Affective Neuroscience, Linköping University, Linköpings Universitet, 58183 Linköping, Sweden; 5 Department of Neurology, University Medical Center, Johannes Gutenberg-University, Langenbeckstr. 1, 56101 Mainz, Germany; 6 Department of Neuroradiology, Medical University Innsbruck, Innrain 52, 6020 Innsbruck, Austria

**Keywords:** CT afferents, Small fiber neuropathy (SFN), Pain perception, Pain inhibition, Spinal cord, fMRI

## Abstract

**Background:**

Human, hairy skin contains a subgroup of C-fibers, the C-low threshold mechanoreceptive afferents ((C-LTMR) C-tactile or C-touch (CT) fibers) that are linked with the signaling of affective aspects of human touch. Recent studies suggest an involvement of these afferents in the modulation of pain in healthy volunteers. Small fiber neuropathy (SFN) is associated with a damage of C-fibers. Therefore, an impairment of C-LTMRs can be assumed. We aimed to elaborate a possible role of CT-afferents in pain modulation by investigating healthy volunteers and SFN-patients.

**Methods:**

Experiment I: 20 SFN-patients (12 women, median age 52.0 years) and 20 healthy controls (14 women, median age 43.0 years) participated in this prospective fMRI and psychophysical study. Heat-pain (HP), CT-targeted touch (slow brushing) and HP combined with CT-targeted touch were applied in randomized order to the left shank in a block design. The participants rated pain intensity on a visual analogue scale. Experiment II: We investigated a possible impact of pain intensity on CT induced pain modulation (10 healthy participants). The intensity of HP stimulation was chosen to induce pain intensity 50/100 (NRS). HP stimulation was applied with and without CT-targeted touch.

**Results:**

Experiment I: CT-stimulation was sufficient to reduce heat pain in healthy participants (*p* = 0.016), but not in SFN-patients. HP induced pain intensity was significantly higher (32,2 vs 52,6) in SFN-patients. During HP, bold responses in pain associated areas were observed in both groups. Additional CT-stimulation elicited no significant difference of bold responses compared to HP. Experiment II: In healthy volunteers, we reproduced a significant reduction of HP intensity by CT-stimulation (*p* = 0.038).

**Conclusions:**

CT input seems to be sufficient to modulate pain, independent of intensity of the pain stimulus. As a prerequisite, the CT fibers have to be intact as in healthy volunteers. If CT fibers are impaired – as in SFN -, CT-targeted touch does not modulate pain intensity. The location of CT-induced pain modulation might be attributed to the level of the dorsal horn since the cortical activation pattern of heat pain with and without CT-targeted touch did not differ in healthy subjects and in SFN-patients.

## Background

In human hairy skin, pleasant touch is signaled by Aβ afferents and a subgroup of C fibers, the C-low threshold mechanoreceptive (C-LTMR; CT = C touch or C tactile) afferents. Those fibers were first detected using microneurography of the infraorbital nerve [1], but were then found to be distributed more widely in the arm and leg [2]. They do not appear in glabrous skin [2, 3]. Characterization of C-LTMR revealed that these afferents react to low mechanical indentation forces (<5 mN) [4] and to slowly moving stimuli [5, 6] as in gentle stroking. The pleasantness of touch is positively correlated with the firing rate of these fibers [6]. In mice, the cell bodies of the C-LTMRs are randomly distributed in the dorsal root ganglia, C-LTMRs terminate in Lamina II of the dorsal horn accompanied by Aδ and Aβ afferents [7]. They enter the lamina I/II spinothalamic pathway up to the ventromedial posterior thalamic nucleus [8, 9].

In humans, the main brain areas receiving C-LTMR information belong to the somatosensory system and affect processing brain networks like the contralateral posterior insular cortex [10] or the medial prefrontal cortex [11]. The intensity of CT targeted touch is encoded in the primary and secondary somatosensory cortex (S1 contralateral, S2 bilateral), whereas the pleasantness is encoded in the pregenual anterior cingulate cortex [12]. C-LTMRs also activate regions involved in reward processing (putamen and orbitofrontal cortex [13, 14]) and in processing of social stimuli (posterior superior temporal sulcus [15–17]).

Animal experiments indicate that CT activation reduces pain, but the precise anatomical localization of this phenomenon is still unknown [18–20]. In humans, slow and gentle touch is supposed to ease pain [21]. Recently a modulation of laser evoked pain by CT targeted touch on the contralateral extremity has been reported. The effect of CT targeted touch (attenuating/ increasing pain) was dependent on the attachment styles (attachment anxiety/ attachment avoidance) of the participants [22]. It needs to be further elucidated, whether an inhibition of nociceptive neurons by C-LTMRs exists in humans and additionally, where this is being processed. An ideal model to answer this question is the investigation of small fiber neuropathy (SFN) patients. SFN is defined as a neuropathy that exclusively affects the thin sensory (C and Aδ fibers) nerve fibers [23]. It is confirmed by thermal testing and skin biopsy. Although C-LTMRS cannot be easily tested, it can be assumed, that C-LTMRs as a subgroup of C fibers should be impaired as well.

In the present study, we examined healthy volunteers with intact C-LTMRs and SFN patients with supposedly impaired C-LTMRs to investigate the impact of C-LTMR signaling on pain modulation in humans. We compared heat pain intensity with and without CT targeted touch (gentle stroking with a soft brush) in both groups. CT targeted touch was proposed to attenuate pain in healthy volunteers and to a lesser extend in SFN patients. To search for possible CNS mechanisms, we performed fMRI.

## Methods

### Participants

Twenty patients with SFN (12 women, 8 men, median age: 52 years, age range: 35–71 years) and 20 healthy controls (14 women, 6 men, median age: 44 years, age range: 30–75 years) were included in the study. All participants were fully right handed according to a modified handedness score [24].

In all SFN patients, the medical history was recorded and a thorough neurological examination was performed. SFN was diagnosed according to the diagnostic criteria by Hoeijmakers et al. [25]. In brief, all patients suffered from length dependent neuropathic pain in the feet and lower legs. In all patients, cold (CDT) and warm detection thresholds (WDT) were determined using quantitative sensory testing (QST). Every SFN patient underwent a skin biopsy. The intraepidermal nerve fiber density (IENFD) in skin punch biopsies was analyzed according to the recommended guidelines to confirm the diagnosis of SFN [26, 27]. Electroneurography was performed to exclude large fiber involvement. SFN was diagnosed if the patients presented with typical clinical features [25], pathological thermal detection thresholds as well as decreased IENFD. Patients were excluded from the study if they showed abnormalities in the clinical neurological examination especially in vibration detection thresholds or pathological values in the motor and sensory nerve conduction studies. Patients that presented with allodynia were also excluded from the study since CT targeted touch would elicit pain in these patients [28].

In healthy volunteers, the medical history, the clinical neurological examination as well as the thermal testing had to be unremarkable.

All participants gave their informed written consent according to the latest revision of the Declaration of Helsinki. The study was approved by the local ethics committee of the Justus-Liebig-University, Gießen (105/11).

### Skin biopsy

In SFN patients, skin punch biopsies (3 mm) were taken 10 cm above the malleolus lateralis in the innervation territory of the sural nerve and the tissue samples were analysed according to the recommended guidelines [26, 29]. In brief the tissue samples were fixed in 2% Zamboni solution and cut into 50 μm thick slices. Immunofluorescence staining was performed at free floating sections with antibody against PGP 9.5 (rabbit polyclonal anti-PGP 9.5 1:1000; zytomed) and a second antibody (goat anti-rabbit Alexa Fluor 488, 1:1000; invitrogen). In a minimum of six sections the intraepidermal nerve fibers (IENF) were analysed using a Leica Immunofluorescence Microscope DM 2000 at magnification 400fold. IENFD was calculated as the mean of IENF per millimetre.

### Cold and warm detection thresholds

All participants were tested for cold and warm detection thresholds on the dorsum of the left foot. The exact procedure has been already described elsewhere [30]. In brief, warm and cold detection thresholds were determined using a TSA 2001-II (MEDOC, Israel; contact area of the thermode: 9 cm^2^; baseline temperature: 32 °C; slope: 1 °C/s). The mean threshold temperature of 3 consecutive measurements was calculated.

### Experiment I: stimulation protocol

**Fig. 1:**
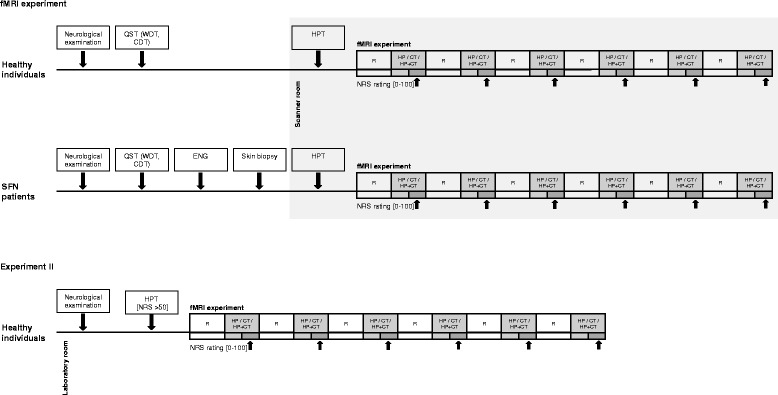
Sequence of the neurophysiological pre-investigations (warm detection threshold (WDT), cold detection threshold (CDT), heat pain threshold (HPT), electroneurography (ENG)) and of the fMRI experiment (rest (R) alternating with the conditions “CT touch” (CT), “heat pain” (HP) and “heat pain with CT touch” (HP + CT), which were performed in randomized order) is demonstrated for both groups. The dark grey scaled boxes indicate the rating periods of pain intensity (on NRS), onsets are marked by black arrows. The big grey box demonstrates the tests performed in the scanner room

During fMRI, three different conditions were applied at the left lower leg in a randomized order: 1. heat pain only, 2. heat pain and CT-targeted brushing and 3. CT targeted touch only. The heat pain elicited pain was rated on a computerized rating scale that was presented via a projector. Each condition was applied for 10 s alternating with rest for a total of 6 times each. The heat pain- and tactile stimulus were synchronized to the MR-images via an electrical trigger from the scanner. The trigger has been linked to a matlab-based program showing a time scale, which has been presented on a screen in the scanner room. The screen could be seen inside (CT stimulation) and outside (heat pain stimulation) of the scanner room. The timescale started with the sequence “rest” and a countdown signalizing stimulus onset, which has been additionally marked as a color change, so that the investigators were primed for the final optical trigger to start the stimulation manually. The rating period started 5 s after stimulus onset and was presented on the same screen. See Fig. 1. When an error of timing occurred, the sequence has been excluded.

We applied all conditions in randomized order to minimize order effects, all participants were blinded for the expected pain inhibition by CT stimulation. Participants were unaware of the aims of the study. They were only informed that central activation patterns and pain intensity during heat pain and touch are investigated in various combinations.

#### Heat pain stimulation

Heat pain thresholds (HPT) were determined in all participants at the left foot within the L5 dermatome. The mean HPT was calculated from 3 consecutive measurements according to the method of limits (for detail see: [31]. The individual heat pain threshold was determined directly before the subject entered the scanner, to obtain stable heat pain intensities the applied temperature was HPT + 1.5 °C. The intended pain intensity was characterized as “painful but bearable”. Heat pain stimulation was carried out at the participants left foot.

#### Tactile stimulation

Repetitive strokes with a soft painter’s brush (2 cm wide, stimulation force 0.8 N; stimulation velocity 3 cm/s) were applied in proximal to distal direction on the left lower leg (CT targeted touch). The stroking was performed over a length of 30 cm with a velocity of 3 cm/s on the lateral lower leg in the area of hairy skin in the dermatome L5. To avoid inter-investigator variability stroking was always performed by the same investigator (KH).

### Pain rating

Subjects were asked to quantify the heat derived pain intensity on a numeric rating scale (NRS; 0–100) with the anchors 0 “no pain” on the left end and 100 “worst pain imaginable” on the right end. The rating started 5 s after the beginning of each trial and lasted for 5 s. The computerized NRS was presented via a projector. Pain ratings were assessed by operating a fiber optic response pad (Current Designs, Inc., Philadelphia, USA) which was connected to a computer. The pad was held in the dominant right hand by all subjects. The handling of the NRS was practiced before the scanning. See Fig. 1.

### fMRI

#### Data acquisition

Functional and anatomical MR scans were performed in a 3 Tesla MR Scanner (Siemens Magnetom Verio, Siemens Medical Systems, Erlangen, Germany) using the 8 channel standard head coil. For structural images, a T1 weighted sequence (TR 1900 ms, TE 2.45 ms, bandwidth 170 Hz/Pixel, TI 900 ms, flip angle 9°, FOV 176 × 512 × 512 mm, orientation: sagittal, voxel size 1 × 1 × 1 mm) was acquired. Functional images consisted of EPI sequences (TR 2800 ms, TE 30 ms, bandwidth 2232 Hz/Pixel, flip angle 90°, FOV 240 × 240 × 120 mm, orientation: T > C-21.4, 40 slices, voxel size 3 × 3 × 3 mm^3^, TA 5:43).

#### Data processing

For data processing, we used Statistical Parametric Mapping (SPM8, Welcome Department of Cognitive Neurology, London, UK). Prior to first level analysis, the images underwent preprocessing. This included realignment to the mean functional image (b-spline interpolation), segmentation of the structural image and normalization into Montreal Neurological Institute (MNI) space. Data were smoothed with full-width and half maximum (FWHM) of 6 mm of the Gaussian smoothing kernel.

A first level analysis was performed for each subject individually using the general linear model with a high-pass filter with a cutoff of 128 s. Contrasts were defined for the different stimuli (heat pain and heat pain combined with CT-targeted touch) vs rest. Subsequently mixed-effects group analyses were performed for healthy subjects and SFN patients separately with a one sample t-test. Additionally, two sample t-tests were used for the evaluation of group differences for the conditions “heat pain” versus “heat pain and CT targeted touch” between healthy participants and SFN patients.

Supplementary, we performed a region of interest (ROI) analyses using the small volume correction feature of SPM. As ROIs we chose the projection sides of CT fibers (insula, medial prefrontal cortex (MPFC) and pregenual ACC). The mask for the insulas were derived from the wfu pickatlas (human atlas, TD labels) for insula bilaterally. For masking the MPFC and pregenual ACC spheres with a diameter of 10 mm were placed according to Liljencrantz and Björnsdotter (medial frontal gyrus, MNI coordinates 11, 52, − 6; − 11, 52, − 6; [11]) and Case 2016 (pregenual ACC, MNI coordinates − 8, 50, 6; 8, 50, 6; [12]). These regions were saved as one image and used as inclusive mask for an additional ROI analysis.

Analysis was performed on a voxel-level and corrected for multiple testing by random field theory with a family-wise error rate of α = 0.05.

For anatomical labelling, FSL based atlases were used (Harvard-Oxford Cortical Structural Atlas; Harvard-Oxford Subcortical Structural Atlas; Juelich Histological Atlas (http://fsl.fmrib.ox.ac.uk/fsl/fslwiki/Atlases)).

For visualization of the results, we used a standard template (SPM 8 template avg152T2), only showing significant activations.

### Experiment II

In the second experiment, we investigated 10 healthy individuals in our laboratory rooms. “Heat pain” and “heat pain with CT targeted touch” were applied to the left foot (within the L5 dermatome), CT stimulation was performed on the left shank by slow brushing (3 cm/s), according to the setup in our first experiment during fMRI. The stimulations were applied in a randomized order lasting 10 s each with 10 s of rest in between stimulations. Heat pain stimulation was chosen to elicit pain intensities >50 on a NRS (0–100; anchors 0 “no pain” and 100 “worst pain imaginable”). The rating started 5 s after the beginning of each trial and lasted for 5 s. NRS rating was performed with the same computerized NRS which we used during the experiment in the scanner room, but was presented on a laptop. Pain ratings were delivered by pressing a computer key.

### Statistics

Psychophysical data were analyzed using the SPSS Statistics (IBM, Version 23.0 for Windows) software package. Kolmogorov-Smirnov tests of normality were run for all data sets and parametric or non-parametric statistics were used accordingly as described in the experiment-specific results. All values are given as medians and interquartile range (IQR) in the case of a non-normal distribution and as means ± standard deviation (SD) in the case of a normal distribution. Values were considered significant if p<0.05.

## Results

### Skin biopsies

The mean intraepidermal nerve fiber density (IENFD) in all SFN patients was decreased compared to normal control values (mean IENFD: 2.46 (SD 1.65); norm: 4.0 to 7.8 [27]).

### Cold and warm detection thresholds (CDT and WDT)

SFN patients had significantly higher warm detection thresholds (SFN patients: median 43 °C, IQR 5.3; healthy participants: 39.8 °C, IQR 6.3; p<0.001) and lower cold detection threshold (SFN patients: median 24.1 °C, IQR 5.7; healthy controls: median 29.0 °C, IQR 2.9; *p* = 0.003). These findings support the impairment of Aδ- (CDT) and C-fibers (WDT) in accordance with diagnosis of SFN.

### Heat pain thresholds (HPT)

SFN patients had significantly higher heat pain thresholds compared to the controls (HPT healthy participants: median 46 °C, IQR 3; HPT SFN patients: median 47 °C, IQR 1; *p* = 0.045).

### Pain ratings during fMRI

**Fig. 2:**
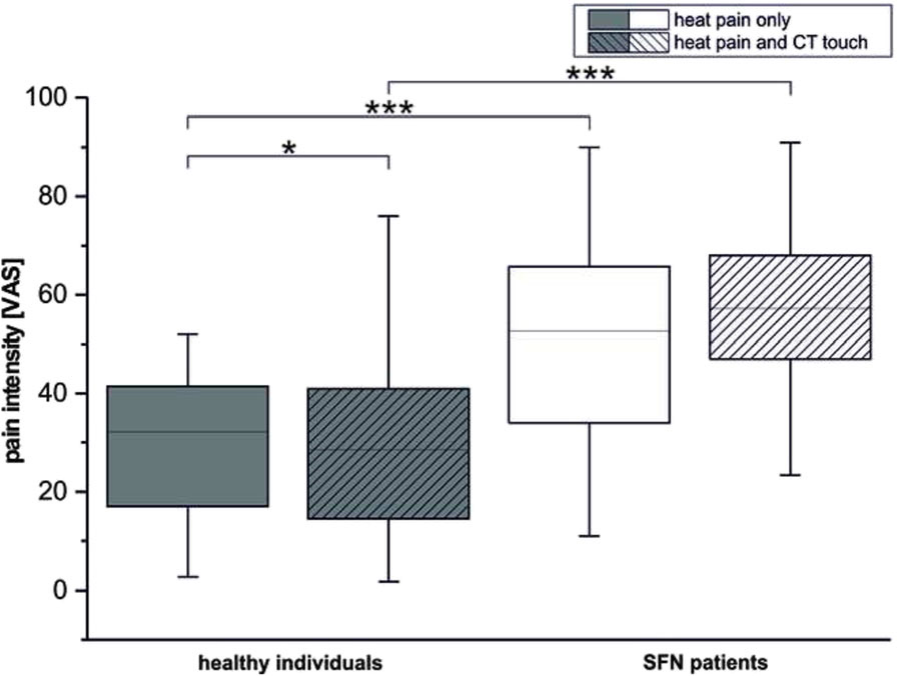
Visual analogue scale (NRS) ratings for the conditions “heat pain only” and “heat pain combined with CT stimulation” in healthy subjects and SFN patients. CT stimulation significantly reduced of heat pain intensity in healthy subjects (*p* = 0.016) but not in SFN patients (* *p*≤0.05, ** of p≤0.01, *** of p≤0.005). The overall rating of pain intensity for both conditions was significantly higher in the SFN patients (heat pain only: *p* = 0.005; heat pain combined with CT targeted touch: p<0.001)

In healthy subjects, heat pain intensity was significantly reduced by the addition of CT targeted touch (NRS heat pain only: mean 32.2, SD 22.35; NRS heat-pain and CT targeted touch: 28.6, SD 21.86; *p* = 0.016, paired t-test). For details see Fig. 2.

In SFN patients, heat pain intensity was not altered by the addition of CT targeted touch (NRS heat pain only: mean 52.6, SD 20.2; NRS heat-pain and CT targeted touch: 57.2, SD 23.08; *p* = 0.139, paired t-test). For details see Fig. 2.

Pain ratings for the conditions heat pain (*p* = 0.005) and heat pain with CT stimulation (p<0.001) were significantly higher in SFN patients compared to controls. For details see Fig. 2.

### fMRI

#### Bold responses for “CT targeted touch” only

**Fig. 3:**
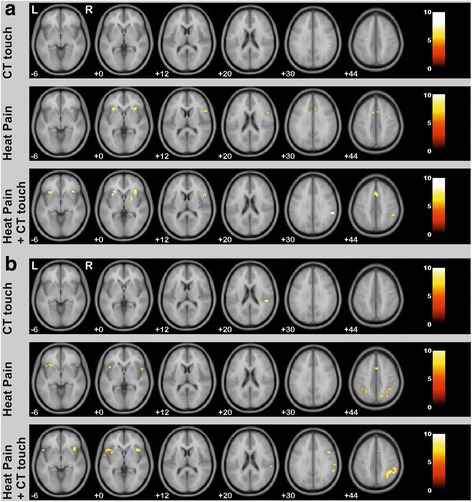
fMRI images for **a** healthy subjects and **b** SFN patients, showing neuronal activation for the main effect of “CT touch”, “heat pain only” and “heat pain combined with CT targeted touch”. The right side of the images corresponds to the right hemisphere. Bold responses are shown with FWE correction, *p*<0.05 and cluster size >3 voxel. Results are shown on SPM template avg152T2

CT targeted touch in healthy subjects showed bold responses in the left postcentral gyrus and the right parietal operculum (supramarginal gyrus). In SFN patients, CT-targeted touch resulted in activation of the right parietal operculum (supramarginal gyrus). For details see Fig. 3 and Table 1.

**Table 1:** Summary of bold responses for “heat pain only”, “heat pain combined with CT targeted touch” and “CT touch”, versus control condition showing MNI coordinates, cluster size and peak T-value for healthy subjects (A) and SFN patients (B)

H	Brain region	MNI coordinate (x, y, z)	cluster size	peak T-value
**A. Bold responses (healthy subjects)**				
Heat pain				
R	Insula (ant)	33, 23, 1	25	10.0226202
R	Middle frontal gyrus, BA 40	45, 35, 25	16	9.40863228
R	Middle frontal gyrus	33, − 1, 55	13	8.20184994
R	Supramarginal gyrus, posterior division	57, − 37, 43	3	7.02497864
R	Inferior frontal gyrus, pars opercularis	48, 14, 13	21	8.32530499
R	Angular gyrus; S2*	45,- 49, 46	9	7.82689619
R	ACC	3, 26, 37	4	7.43887186
R	Paracingulate gyrus	6, 14, 46	58	8.13667107
L	Paracingulate gyrus	-6, 26, 34	8	7.75460911
L	Insula (ant)	− 30, 23, 1	21	10.1233358
L	Lateral globus pallidus*	− 18, 2, 1	4	8.00571537
Heat pain combined with CT targeted touch				
R	Insula (ant.)	36, 26, − 5	63	8.555808067
R	ACC	0, 17, 49	45	7.733303547
R	Putamen	24, 2, 1	8	7.900562286
R	Supramarginal gyrus, BA 40; S2*	57, − 40, 31	19	8.539946556
R	Middle frontal gyrus, BA 40	36, 2, 49	8	7.389503002
R	Anterior intra-parietal sulcus hIP1	42, − 49, 46	10	7.324296474
R	Inferior frontal gyrus, BA 47	42, 20, − 11	3	7.169946671
R	Cerebellum	30, − 58, − 32	24	7.958759308
L	Insula (ant)	− 30, 23, − 2	38	8.298020363
L	Supramarginal gyrus, BA 40	− 42, − 34, 34	5	7.888269424
CT targeted touch				
R	Parietal operculum	48, − 34, 31	3	7.85348225
L	Postcentral gyrus	− 57, − 25, 34	3	6.73631382
**B. Bold responses (SFN patients)**				
Heat pain				
R	Insula (med.-ant.)*	42, 17, − 2	7	7.24895525
R	ACC	3, 11, 46	42	8.66720772
R	Angular gyrus, BA 40; S2*	45,-46, 49	30	10.2263985
R	Precuneous cortex	15,-67, 43	20	8.95573997
R	Lateral occipital cortex	33, − 67, 40	8	8.75775337
R	Lateral occipital cortex	33, − 58, 46	18	8.02260399
R	Broca’s area, BA 44	54, 11, 1	4	8.00252247
R	Cerebellum, anterior lobe	27, − 55, − 20	4	7.94967175
R	Visual cortex, BA 17	18, − 97, 4	4	7.3528986
L	Insula (ant.)*	− 27, 20, − 5	5	8.33598518
L	Superior parietal lobule	− 30, − 52, 49	40	8.32214737
L	Anterior intra-parietal sulcus	− 36, − 43, 37	3	7.88397264
L	Supramarginal gyrus	− 51, − 37, 46	5	7.11958122
L	Frontal operculum	− 42, 17, − 2	13	9.18519974
L	Cerebellum, posterior lobe	− 27, − 62, − 29	42	10.7323313
L	Cerebellum, posterior lobe	− 6, − 76, − 23	22	8.34745884
L	Inferior cerebellum*	− 24, − 61, − 50	21	10.6803532
Heat pain combined with CT targeted touch				
R	Supramarginal gyrus, BA 40; S2*	42, − 46, 46	168	9.750800133
R	Insula (mid-ant)	39, 17, − 2	48	9.569821358
R	Parietal operculum	60, − 28, 22	13	9.502679825
R	Precentral gyrus; S1*	48, 11, 31	14	8.683162689
R	Paracingulate gyrus	9, 14, 40	9	7.428187847
R	Cerebellum, anterior lobe (culmen)	9, − 49, − 17	3	6.953028679
R	Cerebellum, anterior lobe (culmen)	30, − 49, − 26	28	8.335325241
R	Broca’s area BA44	48, 14, 16	4	7.146142006
R	Visual cortex, BA 17	18, − 79, 10	4	6.931876659
L	Frontal operculum	− 48, 17, − 2	40	8.466537476
L	Paracingulate gyrus, premotor cortex BA 6	0, 8, 52	11	7.569615841
L	Precentral gyrus	− 57, 5, 4	8	9.12651825
L	Superior parietal lobule, anterior intra-parietal sulcus; S2*	− 36, − 49, 46	3	7.223741531
L	Supramarginal gyrus, postcentral gyrus	− 42, − 34, 4	12	8.600948334
L	Cerebellum, posterior lobe	− 33, − 64, − 44	12	8.533157349
L	Cerebellum, posterior lobe (Pyramis)	− 9, − 76, − 29	15	9.127865791
L	Cerebellum posterior lobe (Uvula)	− 21, − 67, − 26	6	7.966376305
L	Cerebellum, anterior lobe	− 36, − 55, − 29	32	8.700092316
CT targeted touch				
R	Parietal operculum	48, − 31, 19	8	10.0452766

All activations are shown with p (peak, FWE-corr) < 0.05 and cluster size ≥ 3

*anatomical correspondence in SPM using template

All activations are shown with *p*<0.05 (peak, FWE-correction) and cluster size≥3. Regions marked with * are named after their anatomical correspondence in SPM using the template avg152T2

No significant difference of bold responses could be detected between healthy controls and SFN patients in the condition “CT targeted touch” only.

#### Bold responses for “heat pain” only

In healthy subjects, the heat pain stimulus evoked activations of areas known to be associated with pain processing: anterior insular cortices bilaterally (right 33,23,1; T 10.0; left − 30,23,1; T 10.1) and the anterior cingulate cortex (cluster peak 9,20,31; T 7.2). For details see Fig. 3 and Table 1. In SFN patients the condition “heat pain” also evoked activations in cortical areas associated with pain processing (e.g. anterior cingulate cortex bilaterally (cluster peak at 3,11,46; T 8.7), the insular cortex bilaterally (right anterior-middle insular cortex: 42,17,-2; T 7.2 and left anterior insular cortex: − 27,20,-5; T 8.3). For details see Fig. 3 and Table 1.

No differences in the cortical activation could be detected between healthy controls and SFN patients for the condition “heat pain” only.

#### Bold responses for “heat pain and CT touch”

In healthy volunteers, heat pain and CT targeted stimulation evoked significant bold responses in the anterior insula bilaterally, the ACC and the supramarginal gyrus. Right hemispherical activations correspond to the putamen, frontal opercular cortex, middle frontal gyrus, anterior intra-parietal sulcus, inferior parietal lobule, inferior frontal gyrus and activation of less specified brain regions. There were no supplementary bold responses in the left hemisphere. For details see Fig. 3 and Table 1.

In SFN patients, heat with CT touch elicited bilateral bold responses of the insula, ACC, supramarginal gyrus, the opercular cortex and the anterior intra-parietal sulcus. In addition, the right paracingulate gyrus showed a significant bold response. For details see Fig. 3 and Table 1.

There was no difference of bold responses comparing the conditions “heat pain only” and “heat pain with CT targeted touch” in both groups.

A sensitive ROI analysis for all performed tests detected no additional bold responses in the predefined areas (insula, medial frontal gyrus and pregenual ACC).

### Experiment II

Since heat induced pain intensity was higher in SFN patients compared to our controls, we performed an additional psychophysical experiment (experiment II) in healthy subjects to investigate a possible impact of pain intensity on CT mediated pain modulation. The heat stimuli were set to induce pain intensity NRS 50 from 100 (applied temperature median 47.0 °C, IQR 3.25). With this setup, we reproduced the significant reduction of heat pain by CT stimulation despite high pain intensities (NRS heat pain only: mean 55.9, SD 15.26; NRS heat-pain and CT targeted touch: 48.4, SD 16.97; *p* = 0.038, paired t-test).

## Discussion

We provide psychophysical evidence that C-LTMR activation modulates experimental heat pain in humans. This notion is largely independent of the stimulus induced pain intensity. In SFN, in which CT fibers are presumed to be impaired, CT activation does not alter heat pain. Since the thick myelinated Aβ fibers were intact in both groups, these afferents were assumed to be less involved in pain reduction. The mechanisms how CT modulated pain relief is being processed remain unclear. We did not detect significant differences in cortical activation patterns between heat stimulation with and without CT targeted touch.

### Pain modulation

Heat pain intensity was significantly reduced by CT targeted touch in healthy volunteers. This finding holds true for different pain intensities. It is known from animal experiments that C-LMTRs have pain inhibiting capacities [19]. Our results show that this can also be confirmed in humans. Thus, C-LMTRs are not only important for the mediation of pleasantness [6, 10] and social aspects of touch [32] but also for the modulation of heat pain under physiological conditions. This assumption is supported by the missing effect of brushing hairy skin in SFN patients with probable C-LTMR degeneration.

The question arises whether the findings can be attributed to Aβ-afferent activation. We used a velocity and intensity of brushing that was shown to reliably activate C-LMTRs [6] and to induce a feeling of pleasant touch with a poor spatial localization in two patients with a complete Aβ fibre loss [10, 33]. Coding of pleasantness is one of the major functions of CT afferents [6, 10]. Stroking with a velocity of 3 cm/s has also been shown to correlate with the highest pleasantness ratings [33], substantiating a primary activation of CT afferents. Morrison and coworkers investigated patients with hereditary sensory and autonomic neuropathy type V (HSAN V) with a reduction of thin myelinated nerve fibers. The HSAN V patients did not perceive pleasantness of touch by stimulation with a soft brush even though they have intact Aβ afferents [34]. Using this stimulation protocol, we acted on the assumption to mainly observe effects of CT afferents. Moreover, our SFN patients did not show indications of large fibre neuropathy according to the definition of the disease [25]. We performed electroneurography and measured vibration detection thresholds in all patients in order to minimise large fiber involvement. Nevertheless, as we could not directly measure CT fiber activity, we cannot fully exclude possible Aβ fiber involvement in both groups. An example of Aβ fiber mediated heat pain reduction has been reported by Staud et al. with vibro-tactile in healthy individuals and patients with chronic musculoskeletal pain [34].

Very recently published research by Liljencrantz et al. demonstrated that CT targeted touch (using the same stimulation technique as in our study) but not fast brushing (Aβ targeted touch) nor vibration stimuli reduced heat pain intensity in healthy volunteers [35], confirming our argumentation above.

Suffering from chronic neuropathic pain central sensitization can be expected in SFN patients that might lead to altered transmission of sensory input. We would expect soft stroking to increase pain intensity, since light sensory stimuli can enter the pain pathway. Since we did not observe a significant modification of heat pain (neither increase nor decrease) by CT stimulation in the patient group we suggest that rather CT fiber loss than alteration of sensory stimuli by central sensitization causes the unaltered pain perception during stroking.

### Site of pain modulation

The cortical activation patterns elicited by pain combined with “CT targeted touch” did not differ between SFN patients and the control group, even though during “CT targeted touch only” we observed significant activations in brain areas processing somatosensory information. Therefore, the major site of C-LTMR pain modulation might not be the brain, but the dorsal horn. In the original gate control theory, the spinal cord was considered the site were skin perceptions were modulated before entering the cerebral sensory and affective systems [36]. Many histological studies show a large variety of interneurons and synaptic contacts implying multiple connections of C-LTMRs within the dorsal horn. In mice about 25% of the neurons in lamina I and II in the dorsal horn, known to be the projection side of C-LTMRs, are inhibitory interneurons [37]. These histological observations are already indicative for a modulation of afferent input in the dorsal horn of the spinal tract. Lu and Perl showed in rats that C-LTMR signaling reduces nociceptive C-fiber signaling at the level of the dorsal horn in Lamina I and II [19]. They reported distinct but overlapping projection areas within the dorsal horn of C-LTMRs, Aβ-LTMRs and Aδ-LTMRs, further supporting an interaction on the spinal level.

Besides these histo-morphological implications of spinal mechanisms in rodents Mancini et al. presented data with laser evoked potentials in humans, showing a pain relief by touch with von Frey hairs on a segmental level [21]. Recently they added new information, showing, that touch during laser evoked pain changed the laser-evoked potentials (cortical effect) but also the laser evoked blink reflex [38], implying a pain modulation by non-CT targeted touch at the level of the brainstem or below e.g. the spinal cord.

As we did not perform fMRI analysis of the spinal cord, we are not able to report a change of bold responses by CT stimulation there. A further investigation would be needed to examine a spinal activation pattern by CT stimulation and heat pain, even though a possible extinction of counteracting effects might occur as well on the spinal level.

### Limitations of the study

We did not find significant differences in fMRI activation between the condition “heat pain” and “heat pain combined with CT targeted touch” in both groups. Besides the implications arguing for a spinal mechanism of CT modulated pain inhibition, another possible explanation could be lack of statistical power, even though a prior power calculation was positive. During the fMRI session, the participants had to evaluate their intensity of pain and perform a motor task (rating). Therefore, the subtle stimulus of CT activation might be suppressed. But sole CT targeted touch was performed under the same conditions and elicited significant activations of somatosensory brain areas in both groups. We might have achieved a stronger CT stimulation by using a stroking device with skin temperature instead of a brush with neutral temperature [39]. Case and coworkers found different neural processes for coding intensity (posterior insula, primary and secondary somatosensory cortex) and pleasantness of soft touch (pregenual ACC) [12]. These areas are also involved in pain processing [40] which might explain the similarity of the conditions “heat pain” and “heat pain combined with CT targeted touch”. Hypothetically, CT targeted touch reduces the heat pain derived activation of the posterior insular and primary sensory cortex by spinal inhibition, but since these areas are not pain specific and activated by sensory stimuli (e.g. CT targeted touch) as well, they might extinct each other (activation and deactivation in the same region) so that there is no resulting difference of bold responses.

The stimulation protocol has not been fully computerized, we relied on an optical trigger to start the stimulation sequences. Therefore, an inaccuracy of synchronism (manual stimulation and start of the MRI acquisition) needs to be taken into account. Because of the preprocessing (e.g. realignment to the mean) we assume no significant interference.

Pain ratings were significantly higher in the SFN patients group. Obviously, SFN patients, which suffer from neuropathic pain have thermal hyperalgesia. To control for a possible effect of pain intensity on CT mediated heat pain modulation we performed an additional experiment (heat pain with and without CT stimulation) on healthy individuals with higher pain intensities, according to those rated by SFN patients (NRS = 50). We also observed a significant reduction of heat pain by CT stimulation which renders a major effect of the different pain intensity on the MRI findings less likely. Nevertheless, high pain intensities in healthy individuals do not control for possible alteration of pain pathways in SFN patients (e.g. central or peripheral sensitization in SFN patients).

To better control against possible Aβ fiber involvement in pain reduction by soft stroking, we should have performed tests with different stroking velocities and pleasantness ratings. But the present protocol included fMRI analysis, hence we had to obey limited investigation times to overcome movement artefacts.

## Conclusion

This study provides new evidence that CT fibers own the ability to reduce heat pain perception in healthy subjects. In a next step, it needs to be investigated if C-LTMRs are able to reduce other forms of pain, and how this works. This might open an avenue to new therapy concepts in different pain disorders.

## Abbreviations

CDT: Cold detection threshold
C-LTMR: C-low threshold mechanoreceptive afferents
CNS: Central nervous system
CT: C-tactile, C-touch
fMRI: Functional magnetic resonance imaging
HP: Heat pain
HPT: Heat pain threshold
IENFD: Intraepidermal nerve fiber density
NRS: Numeric rating scale
QST: Quantitative sensory testing
ROI: Region of interest
SFN: Small fiber neuropathy
VAS: Visual analogue scale
WDT: Warm detection threshold
